# Supplementary material for: Nonlinear Guided Wave Tomography for Detection and Evaluation of Early-Life Material Degradation in Plates
Source: Sensors (Basel). 2021 Aug 16;21(16):5498. doi: 10.3390/s21165498 (PMC8402285; doi:10.3390/s21165498)
Supplement: Supplementary file 1 [file sensors-21-05498-s001.zip › sensors-1306465-Supplementary.pdf]

Supplementary Material

Table S1. Nominal material parameters for aluminum. Properties representative are as obtained by Gandhi[42], and the relations between these parameters are referred by Jemiolo[22].

**Material**	λ	μ	ν_1	ν_2	ν_3	ρ
Aluminium	54.9 GPa	26.5 GPa	–205.8 GPa	–149.3 GPa	–87.8 GPa	2800 km/m^3

a. ‘ λ ’ and ‘ μ ’ represents Lamé constants.

b. ‘ ν_1 ’, ‘ ν_2 ’ and ‘ ν_3 ’ are Murnaghan’s TOECs.

d. ‘ ρ ’ means mass density.
